# Do you get what you see? Insights of using mAP to select architectures of pretrained neural networks for automated aerial animal detection

**DOI:** 10.1371/journal.pone.0284449

**Published:** 2023-04-24

**Authors:** Mael Moreni, Jerome Theau, Samuel Foucher

**Affiliations:** 1 Department of Applied Geomatics, Université de Sherbrooke, Sherbrooke, Quebec, Canada; 2 Quebec Centre for Biodiversity Science (QCBS), Montreal, Quebec, Canada; Vellore Institute of Technology: VIT University, INDIA

## Abstract

The vast amount of images generated by aerial imagery in the context of regular wildlife surveys nowadays require automatic processing tools. At the top of the mountain of different methods to automatically detect objects in images reigns deep learning’s object detection. The recent focus given to this task has led to an influx of many different architectures of neural networks that are benchmarked against standard datasets like Microsoft’s Common Objects in COntext (COCO). Performance on COCO, a large dataset of computer vision images, is given in terms of mean Average Precision (mAP). In this study, we use six pretrained networks to detect red deer from aerial images, three of which have never been used, to our knowledge, in a context of aerial wildlife surveys. We compare their performance along COCO’s mAP and a common test metric in animal surveys, the F1-score. We also evaluate how dataset imbalance and background uniformity, two common difficulties in wildlife surveys, impact the performance of our models. Our results show that the mAP is not a reliable metric to select the best model to count animals in aerial images and that a counting-focused metric like the F1-score should be favored instead. Our best overall performance was achieved with Generalized Focal Loss (GFL). It scored the highest along both metrics, combining most accurate counting and localization (with average F1-score of 0.96 and 0.97 and average mAP scores of 0.77 and 0.89 on both datasets respectively) and is therefore very promising for future applications. While both imbalance and background uniformity improved the performance of our models, their combined effect had twice as much impact as the choice of architecture. This finding seems to confirm that the recent data-centric shift in the deep learning field could also lead to performance gains in wildlife surveys.

## 1. Introduction

Sound management of animal population relies on regular and reliable surveys [1]. The use of aerial imagery to carry out these surveys has shown to generate more reliable counts than direct visual counting [2] and allows the data to be stored and analyzed by different observers [3] to increase the robustness of the results. Unmanned Aerial Vehicles (UAV), commonly known as drones, add to these benefits by reducing the risks for the researchers and have the potential to significantly reduce the data acquisition costs over time [4, 5]. The main drawback of its extensive use is the large amounts of data it generates, requiring automatic tools to be able to process it [3, 6, 7]. Deep learning, the most promising array of techniques to automatically process these vast amounts of data at the moment [8, 9], is a computer science discipline that focuses on using algorithms built from individual functions stratified along many layers (hence the term deep) that automatically extracts information from the data to solve a given task (learning). The types of problems it is used to solve are extremely varied, from speech recognition [10] to detecting and locating objects in images, known as object detection [11].

The synergy between aerial imagery and object detection has been in the spotlight recently [12–14]. The massive amount of data generated by aerial imagery can be processed automatically by deep learning models [4] whose performance increases with the amount of data they are trained on [15]. This is particularly interesting for animal survey projects in which the necessity of regular acquisitions multiplies the number of images to process and therefore benefits the most from automated ways to generate reliable counts. This recent interest from both the private and academic sector has led to a regular stream of new network architectures improving the state of the art on various standard object detection datasets such as ImageNet [16] or Microsoft’s Common Objects in COntext (COCO) [17]. While the models can often be found with their respective papers, many of these architectures have then been grouped together in sets called model zoos, as in PyTorch [18], Detectron2 [19] and MMDetection [20]. These last two toolboxes are particularly useful in setting up object detection tasks and comparing different models as they allow to seamlessly call different architectures within the same training pipeline. With 50 different architecture baselines, each with various backbone networks, MMDetection offers the largest free collection of models in an integrated toolbox to our knowledge. It also has a thorough and comprehensive library to train and test the models, and an active user base to help with any possible issues. Models are either untrained or pretrained on various datasets, with COCO being the main reference [18–20]. Pretrained models allow to leverage previous training of the deeper layers of the model, that detect simple features, to accelerate the training on new objects of interest. The default training and testing scripts provided allow a practitioner with an annotated dataset to quickly obtain results and therefore save a lot of time by not developing training and testing pipelines themselves. This makes MMDetection a good place to start for someone wanting to implement automatic animal counting. The performance of the many different architectures of these model zoos are given in the metric used in COCO, the mean Average Precision (mAP) [17]. This metric is also the default training metric in the training and validation pipelines in MMDetection.

With so many different models to pick from, many of which have not been used for animal surveys, one would be tempted to lean towards the models with the highest mAP on COCO, which would seem reasonable if the images of interest were very similar to the COCO images. However, aerial animal imagery tends to have significant differences with the COCO dataset. The images are usually quite large (several megapixels), multiple times the size of the images in COCO (in the order of 640x480 pixels). Within them, the animals are seen from farther away than the objects of interest in COCO and are therefore much smaller. They are also much scarcer than their counterparts on COCO, both within images but also in the dataset, resulting in many empty images, containing only background [4, 21]. A significant difference of occurrence of a class over others (including the background) is called class imbalance and can negatively impact the performance of the network in a way that may not have been shown on a more balanced dataset like COCO [22]. Finally, mAP is a metric that averages precision over high values of another metric that quantifies the overlap between the bounding box around the ground truth object and the prediction, the Intersection-over-Union (IoU). In the context of wildlife surveys where the goal is to obtain the most accurate count possible and where the precision of the location is secondary [23], metrics at a fixed IoU such as precision, recall or their harmonic mean, the F1-score seem more appropriate. The F1-score is commonly used to evaluate models performance in this context [4, 21] but some studies also use mAP instead [23, 24]. We are then left to wonder how well the performance on COCO in mAP could predict how the same architecture would perform on an aerial animal survey, and how well would a model trained with mAP perform along an operational metric like F1-score.

Our main focus in this article is to assess the reliability of mAP to select the best architecture to detect and count animals in aerial images. Our methodology also allows us to discuss other aspects of importance in this task such as architecture performance, the impact of data imbalance and background uniformity. To these ends, we trained six pretrained models from MMDetection, three of which have never been used in this context to our knowledge, on two versions of two different datasets of aerial images of red deer (*Cervus elaphus*), one with only images containing deer and the other with both deer and background only images. We then compared the results of an inference based on mAP to a practical output in a scenario of animal counting through the use of F1-score and evaluate whether mAP, on both COCO and on our datasets, can be used as a reliable predictor of performance for animal counting in aerial images. To our knowledge, our study is the first to explore these aspects.

## 2. Methods

### 2.1 Data acquisition

**Fig 1:**
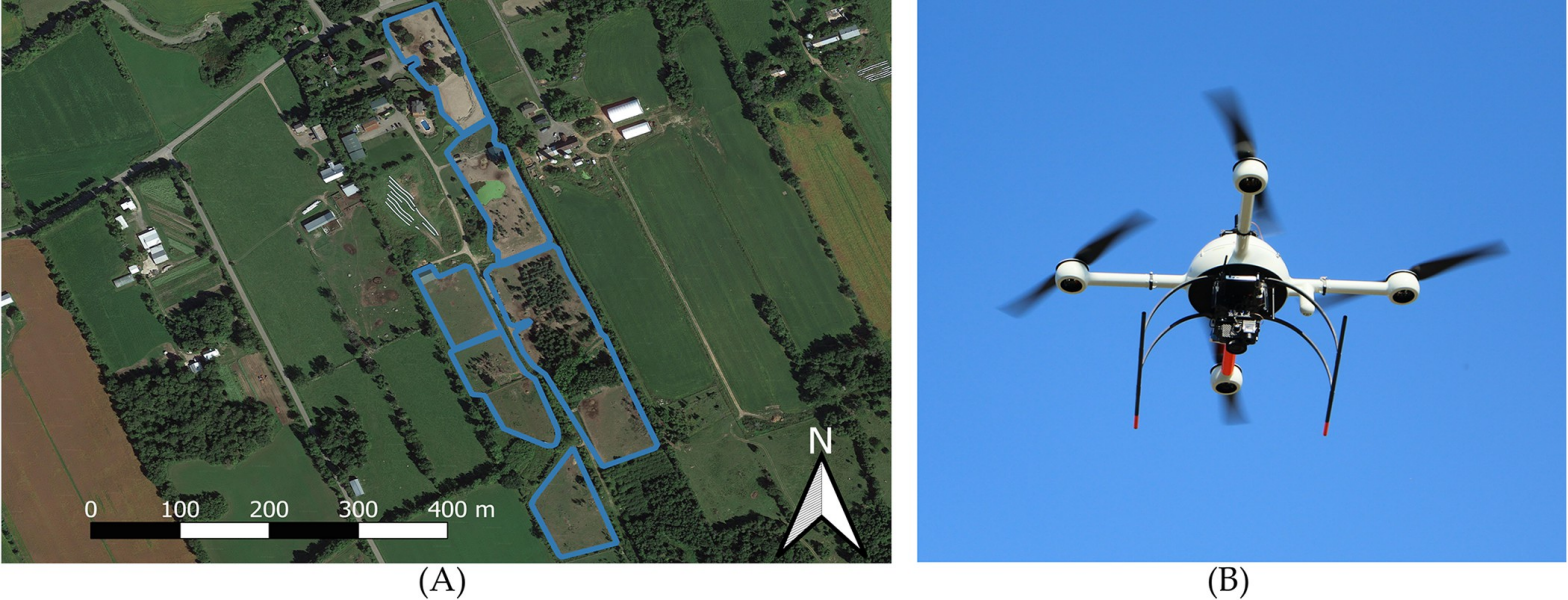
Outline of deer enclosures (in blue) (A) and Microdrone’s md4-1000 UAV (B).

We acquired images of red deer over a deer farm located near Lachute in Quebec, Canada. The high density of animals in the enclosures compared to their natural density in the wild ensured that we would obtain many images of deer in a short time. The flights were carried out with an electrical multirotor UAV, the md4-1000 (Fig 1B) from Microdrones (Berlin, Germany). It was equipped with a Sony RXI RII camera and a 35 mm lens and took RGB images of 7852x5304 pixels. The flights were conducted over a private farm with the full authorization of the owner. The project did not require approval from an animal research ethics committee. Our research did not involve any interaction with the deer. Drone take-offs and landings were carried out away from the enclosures to avoid any possible disturbance.

**Fig 2:**
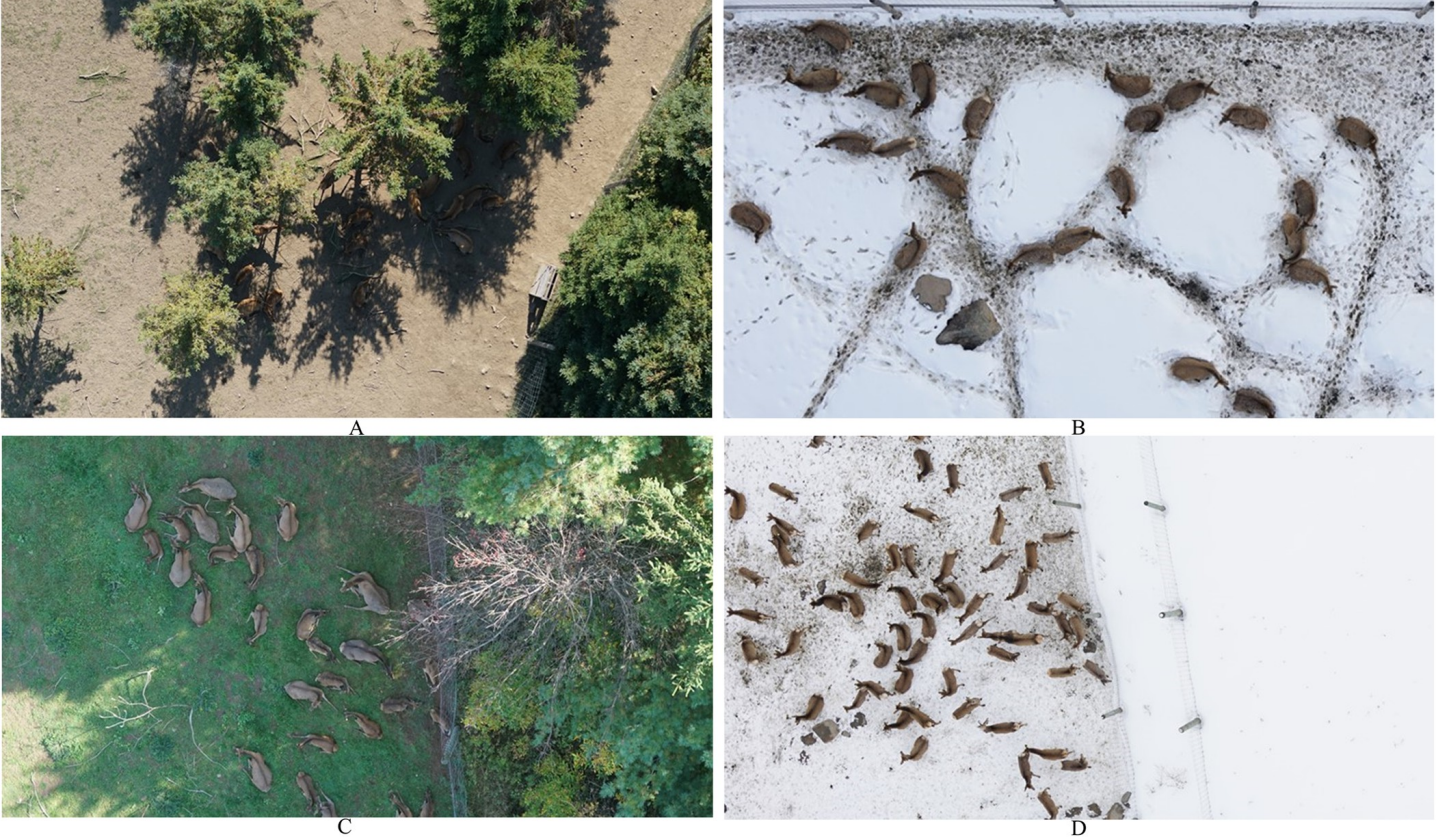
Example of images from the training (top) and validation (bottom) sets of the summer (left) and winter (right) acquisitions.

We acquired two datasets, one in September 2018 and another in March 2019 to have images with and without homogenous snow cover. In the summer acquisition, the deer were in five different enclosures (Fig 1A). The vegetation cover was different in each enclosure, some had a dense coniferous cover and bare soil, others had small patches of deciduous trees and homogenous grass cover (Fig 2). In the winter, the deer were grouped in three of those enclosures. However, the snow was beginning to thaw leaving some bare soil patches, in particular in the areas around the feeding stations where the deer would gather. The flights were conducted at an altitude of 40 meters in the survey mode of the UAV controller application mdMapper, that automatically generates transect lines to cover a designated area. Our images had a Ground Sampling Distance (GSD) of 5 mm. In the summer, we organized the flights per enclosure. In the winter however, the cold temperatures reduced the battery life in a way that it was deemed safer to fly over the three enclosures every time. During both acquisitions, the images were taken every two seconds and had an overlap (frontal and lateral) of 80%.

### 2.2 Data preprocessing

The high overlap between consecutive images within one flight led to very similar successive images. Splitting them randomly between training and validation sets may strongly bias the results by validating on almost the same images as the models have been trained on. To avoid this, we grouped images by flight and generated our training and validation sets according to the flights. This meant that the split between training and validation data was not strictly 60–20% of the total available data.

The annotation of the summer dataset was carried out manually in LabelImg [25] while the winter dataset was annotated with FiftyOne [26] and Computer Vision Annotation Tool (CVAT) [27].

Two versions of each dataset were generated, one with only the positive images, containing at least one deer (posOnly), and another on the whole dataset (wds) on both positive and negative images (background only) (Table 1). This allowed us to test the impact of the addition of many background-only images, thus creating a much higher imbalance between the background and the deer classes.

**Table 1:** Dataset composition in number of images and in number of deer instances for training (train) and validation (val) datasets for the positive-Only (posOnly) and whole-dataset (wds) versions of the summer (S) and winter (W) datasets.

	S_posOnly		S_wds		W_posOnly		W_wds	
	train	val	train	val	train	val	train	val
**Positive images**	328	67	328	67	437	86	437	86
**Negative images**	0	0	1203	426	0	0	1347	158
**Total images**	328	67	1531	512	437	86	1784	244
**Deer instances**	3052	958	3052	958	4652	675	4652	675

### 2.3 Training parameters

#### 2.3.1 Model selection

The training process was carried out with the MMDetection toolbox. Some models in MMDetection were originally developed with libraries other than PyTorch, forcing the MMDetection team to re-implement them. The team tries to match the same performance and the same parameters as the original implementations and make sure to mention any differences between the two.

FRCNNX101 and Libra have been used in animal survey studies and achieved good performances [4, 28]. FRCNNR50 is a milestone in object detection and was expected to perform worse than the other five, more recent architectures. DefDETR was selected because of its transformer-based architecture. Transformers [29] come from the natural language processing field on which they have beaten the previous state of the art architectures and have now been adapted to the object detection task [30]. DefDETR had at the time the highest mAP on COCO of all transformer-based architectures on MMDetection. GFL had a high mAP on COCO as well as an improved focal loss function to deal with dataset imbalance. VFNet had, at the time of the selection, the highest mAP score on COCO of all MMDetection models and a new loss function designed to improve the prediction localization accuracy. We couldn’t find any cases of DefDETR, GFL or VFNet being used to count animals from aerial images at the time of selecting the models for this study.

The six models we used are summarized in Table 2.

**Table 2:** Selected models, their backbones and key features.

Model name	Backbone	Notable features	Number of parameters in millions (M)
**FRCNNR50 [31]**	FRCNN R-50-FPN	Milestone in object detection Cross-entropy loss	41.12 M
**FRCNNX101**	FRCNNX-101-64x4d-FPN	More layers than FRCNNR50 Cross-entropy loss	98.84 M
**GFL ([32])**	FasterRCNN X101-32x6d-dcnv2	Continuous version of Focal loss [33]	53.07 M
**Libra [34]**	FasterRCNN X10164xd-FPN	IoU-balanced sampling, Balanced feature pyramid and balanced L1 loss	99.11 M
**DefDETR [35]**	R-50	Transformer model adapted to computer vision Focal loss	40.8 M
**VFNet [36]**	FasterRCNN X10164xd-FPN	Varifocal loss	90.2 M

We trained our models on the Compute Canada servers, using NVIDIA A100-SXM4-40GB GPUs.

#### 2.3.2 Training schedule and hyperparameters

We opted for a transfer learning approach, starting with pretrained networks and finetuning them on our images. By using this technique to train our models, we reduced the training time compared to if we had been training from scratch. All of our models were pretrained on COCO (and the first stages of the networks are frozen by default in MMDetection). We used the same learning rate schedule for all models: 10 epochs in total with a linear warmup phase of 500 iterations followed by a flat value and a two-step decay at epoch 6 and 8. We found that gradient clipping helped stabilize the training for larger learning rates and therefore opted to apply it to all models.

**Fig 3:**
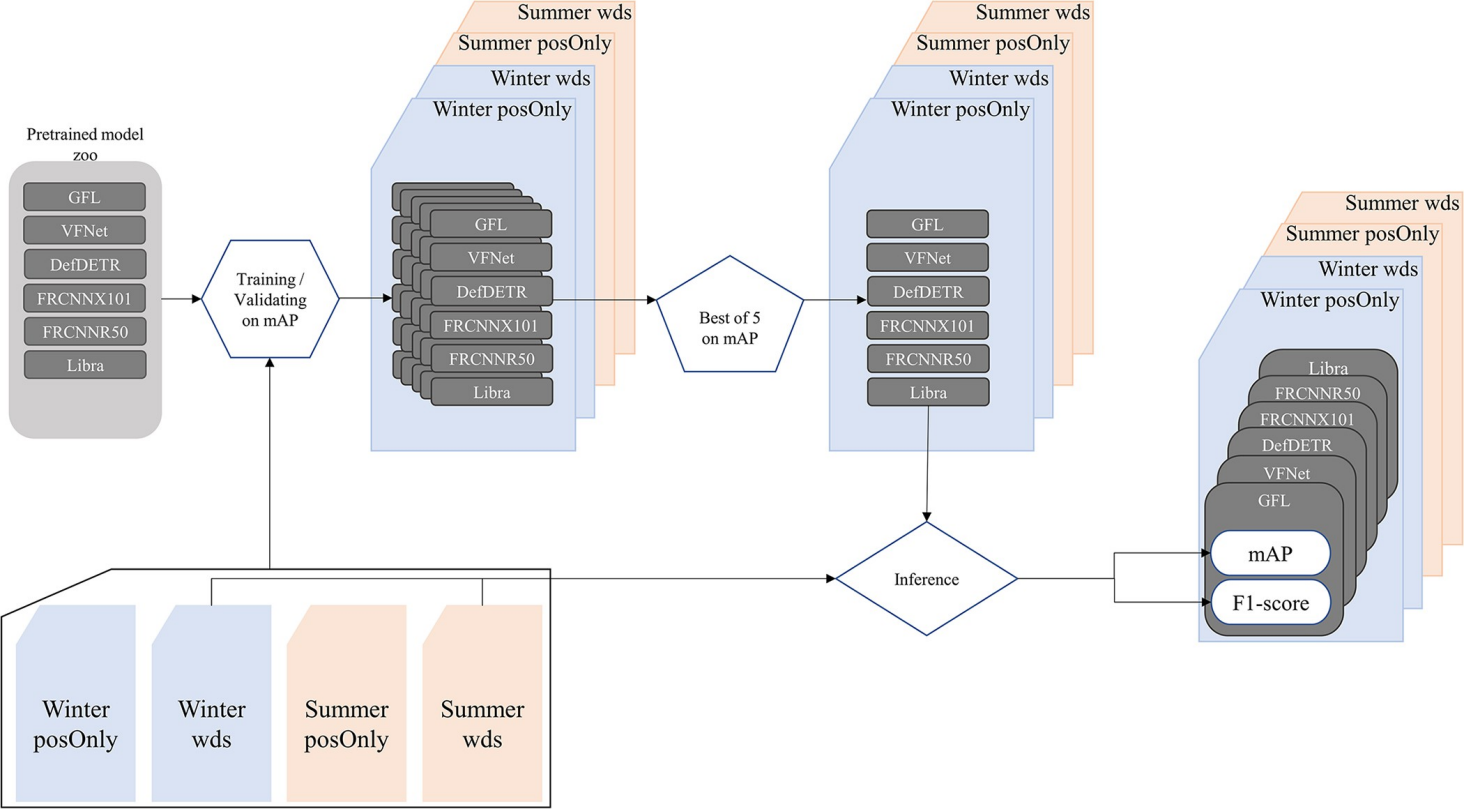
Training and inference methodology for winter (blue) and summer (orange) positive only (posOnly) and whole dataset (wds) datasets.

The learning rates were determined by training each model on each of the four datasets using eight values of learning rate, each one the tenth of the previous one, from 2.5e-1 to 2.5e-8. Even for the wds datasets, the training times were short enough to train each model for 10 epochs to select the learning rate. This allowed us to select a good value of learning rate with minimal effort. The training was set to save the best weights according to the best mAP value found during the validation phase. The learning rate value that produced the best mAP on the validation set was then used to reproduce the training four more times, for a total of five models per architecture and per dataset. We selected the best of those five to use for inference. Because the GPU are randomly allocated on the Compute Canada servers, reaching true repeatability based on seed is impossible. We therefore changed the seed every time and selected the best model out of the five runs. The overall methodology is summarized in Fig 3.

We used random horizontal and vertical flips for data augmentation as well as a resize to 2650*1768 pixels. This size ensured that the images would fit in the GPU’s memory for all the selected models. After the resize, the GSD was of 15 mm. Because the deer would have the same size relative to the distance to the drone, we removed the multiscale augmentations from the data augmentation pipelines found in some architectures. The rest of the training parameters were left to the default values of the model’s configuration file. We used two samples per GPU whenever possible. However, because of the limited resize used on our large images, some models had to be trained with one sample per GPU.

The models trained on the posOnly dataset were run in inference mode on the wds validation set to assess their performance on negative images and compare them to the performance of the versions trained on the wds. Because we are just comparing the validation results along different metrics and the way they direct the model selection in relation to the objectives of animal surveys, we did not include a test set.

Training times were of around 30 minutes on the posOnly datasets and 2h30 minutes on the wds datasets.

### 2.4 Analysis and visualization tools

#### 2.4.1 IoU

**Fig 4:**
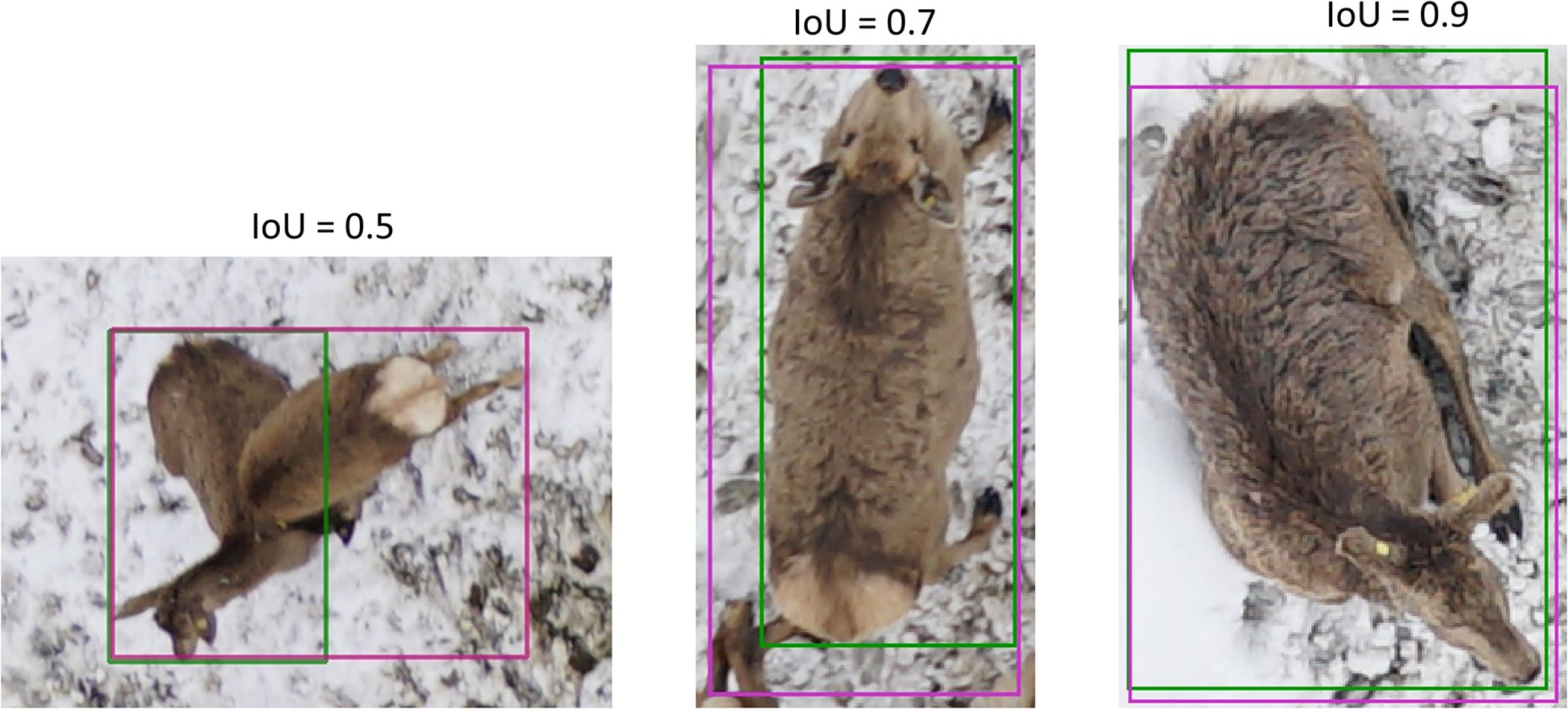
Examples of different IoU thresholds between the ground truth (green) and the prediction (purple).

The IoU is a metric that quantifies how similar two bounding boxes are (Fig 4). It is defined by the ratio of the area of their intersection divided by the area of their union.


$$IoU=\frac{Area(bbox1\cap bbox2)}{Area(bbox1\cup bbox2)}$$ (1)

#### 2.4.2 AP and mAP

The mean Average Precision as used in COCO for a given class is based on the following version of the Average Precision (AP):

$$AP=\frac{1}{101}\sum_{rt\in \{0,0.01,\ldots ,1\}}\;P_{Interpolated}\;(rt)$$ (2)

Where *P_Interpolated_* is the maximum precision at a recall value equal or higher than recall threshold rt, with rt between 0 and 1 at a 0.01 interval [37].

Commonly set at an IoU value of 0.5, the MS COCO challenge uses the mean of the AP over 10 IoU thresholds iou, from 0.5 to 0.95 at a step of 0.05, and then averaged over the of NC number of classes c from the class set C, as its metric of reference:

$${mAP}_{COCO}=\frac{1}{NC}\sum_{c=1}^{NC}\;\frac{1}{10}\sum_{iou\in \{0.5,0.55,\ldots ,0.95\}}\;{AP}_{c,iou}$$ (3)

**2.4.3 Precision, recall and F1-score.** At a given IoU threshold, the Precision describes what proportion of a model’s predictions are indeed correct detections. In other words, how much is the model overpredicting.


$$Precision=\frac{Correct\;predictions}{Total\;number\;of\;predictions}$$ (4)

The Recall describes the proportion of the total number of individuals that the model has correctly detected, or how much is the model missing.


$$Recall=\frac{Correct\;predictions}{Total\;number\;of\;ground\;truths}$$ (5)

The F1-score is a metric that ranges between 0 and 1 that considers both precision and recall as their harmonic mean:

$$F1-score=2\times \frac{Precision\times Recall}{Precision+Recall}$$ (6)

These three metrics can also be calculated at a given confidence score, that allows to filter out the predictions the model outputs but are far away from its internal representation of the class.

#### 2.4.4 Analysis workflow

The predictions resulting from the inference on the wds datasets were exported into Fiftyone to output F1-scores at an IoU of 0.5 for different values of confidence score. The confidence score is a value between zero and one that indicates how confident the model is about a given prediction. By default, all models output predictions above a confidence score of 0.05. At this low confidence threshold, some models generated many low score predictions that would be very tedious to manually remove, but very easy to filter out. In the context of animal surveys, the inference on a dataset must generate the most accurate count possible, one that would require none or very minimal human correction. To that end, it is necessary to decide which minimal IoU threshold value to be considered a correct detection. Similar to the IoU, we also need to select a confidence score threshold so that the number of detections are not skewed by a large number of very low confidence predictions. On our datasets, it seemed that 0.5 confidence score was the point at which most models had the best balance between false positives and false negatives and therefore the best F1-score. We filtered the results of inference to keep the predictions above 0.5 in confidence score then computed the average rank of each model on all four datasets for the F1-score, the mAP and the mAP on COCO from MMDetection and used them to generate Spearman’s correlation coefficient (SCC).

We also generated plots of AP per IoU curves to visualize how the AP would change with higher IoU threshold per model.

## 3. Results

### 3.1 Comparison between mAP on COCO, mAP on our datasets and F1-score

The results of the “best of five” for each model and on each dataset as well as their relative rank are given in Table 3.

**Table 3:** Results of inference on summer (S) and winter (W) datasets for models trained on only positive images (posOnly) and whole dataset (wds) versions of the summer and winter datasets.

	S_posOnly		S_wds		W_posOnly		W_wds		COCO			
Model	mAP	F1-score	mAP	F1-score	mAP	F1-score	mAP	F1-score		rank mAP COCO	average mAP rank	average F1-score rank
**VFNet**	0.77	0.95	0.73	0.95	0.88	0.96	0.87	0.96	0.51	1	2	3
**GFL**	0.77	0.96	0.76	0.95	0.89	0.97	0.88	0.97	0.48	2	1	1–2
**defDETR**	0.62	0.89	0.67	0.93	0.82	0.93	0.83	0.94	0.47	3	5–6	6
**FRCNNX101**	0.71	0.96	0.71	0.96	0.85	0.96	0.85	0.97	0.43	4–5	3	1–2
**Libra**	0.7	0.94	0.7	0.97	0.83	0.89	0.83	0.94	0.43	4–5	4	5
**FRCNNR50**	0.68	0.92	0.68	0.95	0.81	0.96	0.81	0.96	0.38	6	5–6	4
**average**	0.71	0.94	0.71	0.95	0.85	0.95	0.85	0.96	0.45			
**standard deviation**	0.05	0.02	0.03	0.01	0.03	0.03	0.02	0.01	0.04			

Overall mAP results on our datasets, where the lowest score is 0.62, are much higher than on COCO on which the highest score is VFNet at 0.51. On our datasets, the overall best performer is the GFL model, which scores the highest on three out of four datasets on both F1-score and mAP metrics. The worst performing model on our datasets was FRCNNR50 as it is the case on COCO, tied with defDETR even though it is the 3^rd^ best on COCO. Libra had low scores on all three metrics but took the 1^st^ place on the S_wds in F1-score. Whilst FRCNNX101 was 4th on COCO, it matched GFL on average F1-score, but was overtaken by VFNet on mAP. VFNet showed the opposite trend of being close to GFL on mAP but dropping behind FRCNNX101 on F1-score, despite having the highest score on COCO. The SCC confirmed this by showing a very low, albeit positive, correlation of 0.19 between mAP on COCO and F1-score on our datasets.

The SCC showed positive correlations between the mAP and both COCO mAP and F1-score at 0.70 and 0.79 respectively. On our datasets and despite a positive correlation between them, it seems that scoring the highest on mAP doesn’t necessarily imply the same on F1-score and vice versa. Libra and FRCNNX101 both scored in the middle of the range on mAP but were first on F1-score on S_wds and S_posOnly, respectively. Despite GFL being quite ahead of FRCNNX101 on mAP on all four datasets, it ended up tied on F1-score.

### 3.2 Imbalance

The impact of imbalance (with imbalance ratios of 1 positive image for 3.7 and 3.1 negative images for the summer and winter datasets respectively) on our models has been small on average and these small effects would tend to favor both unbalanced datasets. They either perform the same or better, with improvements of 0.01 on mAP in the winter dataset while the average mAP is the same on the summer dataset, and improvements of 0.01 in F1-score for both datasets. The best model overall however, GFL, was impacted slightly negatively by the imbalance and performed better on the summer posOnly dataset.

### 3.3 Background uniformity

The average performance in winter conditions was noticeably higher than on the summer dataset, with an improvement of 0.13 and 0.14 in mAP for the posOnly and wds datasets respectively. This difference was much smaller on the average F1-score, with a 0.02 improvement on posOnly datasets and 0.01 on wds datasets. The differences between posOnly and wds were also smaller on the winter dataset, with five out of six models showing no differences or differences of 0.01 on both mAP and F1-score. The outlier was Libra, who seemed the most impacted (positively) by the imbalance and gained 0.05 points in F1-score on winter wds dataset compared to its posOnly counterpart. The summer conditions showed wider differences between the posOnly and wds datasets, with five out of six models showing differences between the two. Only FRCNNX101 had exactly the same performance on posOnly and wds datasets.

### 3.4 AP per IoU

**Fig 5:**
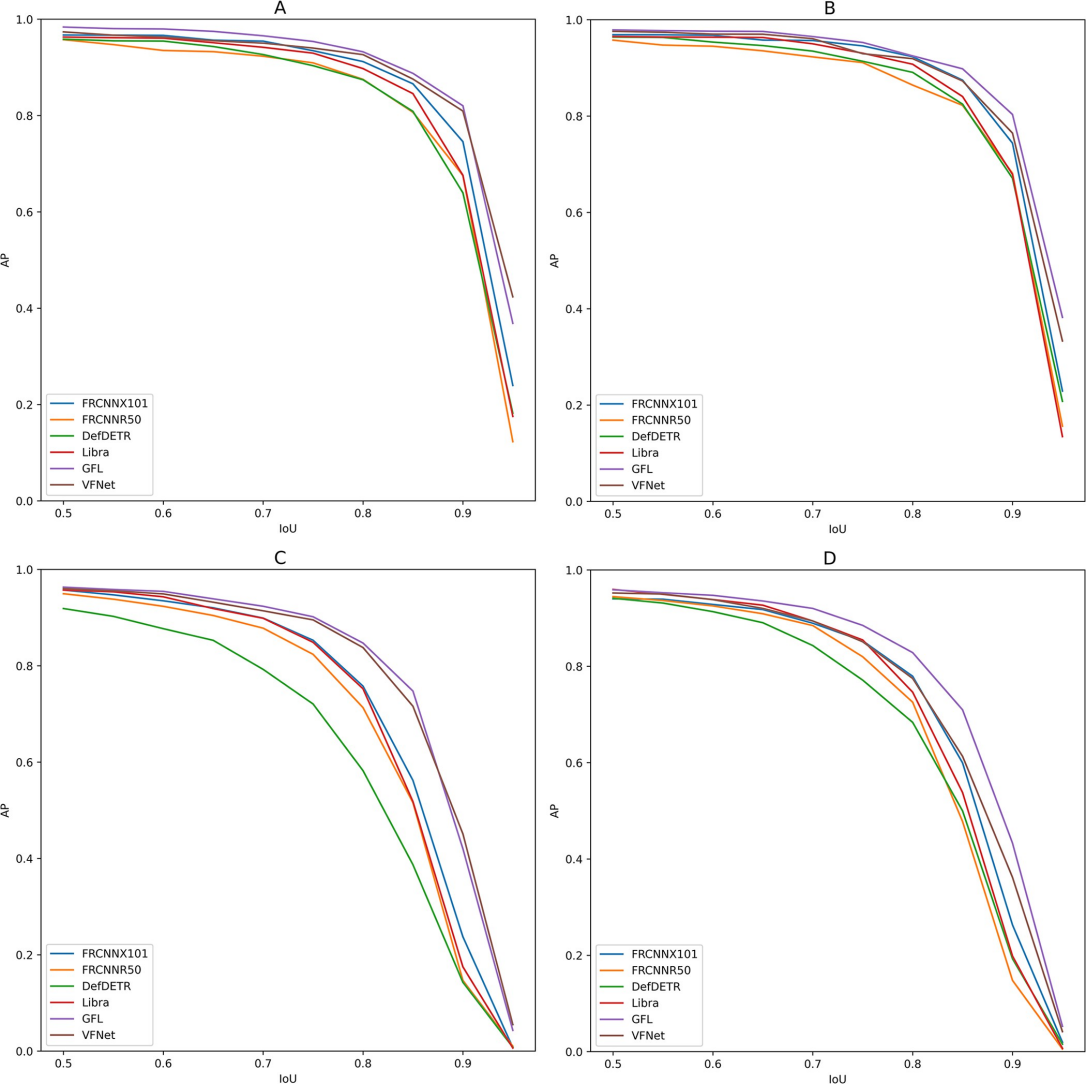
Curves of Average Precision (AP) values per Intersection over Union (IoU) thresholds for each model on our four different datasets: (A) W_PosOnly, (B) W_wds, (C) S_PosOnly, (D) S_wds.

The validation AP per IoU thresholds (Fig 5), show that the AP of all models (apart from defDETR on the summer posOnly dataset) are similar at IoU of 0.5 but the gap between GFL, VFNet and FRCNNX101 and the rest increased with higher values of IoUs. We also note that the performance on the winter dataset at high IoU thresholds is much higher than on the summer dataset. This is especially true for the top performing models, GFL and VFNet, that end with AP of around 0.4 on the winter dataset as opposed to close to 0 on the summer dataset.

## 4. Discussion

### 4.1 Architecture results and comparison

One of our main focus was assessing the reliability of mAP as a way to select a model at the validation step and therefore did not require a test set. As long as the test set is similar to the validation set, we can expect results slightly lower but in the same magnitude as on the validation set. The following comparisons with other studies’ test results will focus on the order of magnitude and trend rather than specific results.

The overall performance of all models with very little hyperparameter tuning was very high on our datasets. The mAP results were much higher than the ones on COCO, showing that a simpler task (one class instead of 80) and higher resolution can lead to better performance. This also means that the differences between our images and COCO’s are not so big as to require much longer training or to unfreeze the deep stage frozen by default in the configuration file. We reach similar conclusions when comparing our results with other aerial survey studies. Delplanque et al. (2021) is the only study to our knowledge to use MMDetection models for aerial animal survey. Despite a lower tuning of hyperparameters and fewer epochs of training, our lower F1-score was 0.27 points higher than their higher score. Such a stark difference in performance is in our eyes caused by the lower GSD we had in our datasets compared to theirs (15mm vs 24mm), coupled to a much simpler task of detecting one species in a controlled environment instead of six in the wild. While we couldn’t find uses of GFL, VFNet or DefDETR in a context of animal survey, the way GFL outperformed Libra, the best model in Delplanque et al. (2021), in our study seems particularly promising for its application in real wildlife survey conditions. Peng et al. (2020) had very high levels of F1-score despite GSD three times bigger (5cm) than ours but with a very similar task, with one species of ungulate in open environments. They trained for ten times the number of epochs we used, on fewer animal instances. Such long training times, despite their use of pretrained networks, may come from the extensive data augmentation they employed. In their study the modified version of FRCNN outperformed their baseline FRCNN and RetinaNet. Our FRCNNX101 model seemed unaffected by our levels of imbalance when it was heavily sensitive to it in Peng et al. (2020), We believe it to be caused by the much higher level of imbalance in their study (around 55 negative images for one positive image compared to around five for one in ours).

Since we used a close to default parameters for models, the size of the bounding boxes was not tuned to fit the size of the deer. DefDETR and GFL are the only two models without anchors and bounding box presets. However, the underwhelming results of DefDETR seem to indicate that it may require longer training times and a better tuning of its parameters compared to the other models. The benefits of learning the bounding box parameters, like DefDETR does, instead of relying on presets of anchor sizes are only worth it if the model reaches or surpasses the performance of its competitors in a reasonable amount of time. While we may need longer finetuning and possibly training time to see whether DefDETR performance could end up reaching the ones of the other architectures we tested, its benefits may not be worth the extra time spent to achieve them. In our context of regular surveys of the same species, the size of the animals on the images is easily predictable, and our nadir angle makes it even more consistent compared to oblique acquisitions. Therefore, a quick calculation of the maximum size of a bounding box would likely improve the accuracy of all our anchor-based models. This led to consistent improvements in Peng et al. (2020) and should be considered in similar cases.

### 4.2 mAP

Based on our experiments, mAP scores on COCO don’t seem to be reliable as a predictor of good counting performance of animals in aerial imagery. The correlation between the mAP on COCO and F1-score on our datasets is very small and would be even closer to 0 if we were to remove GFL from our testing.

The AP per IoU curves show how most of the difference between the top performer and the rest of the models is due to higher AP in higher thresholds of IoU not used in practice. In our experiments, the resolution was extremely high compared to what is used in other aerial surveys, making it possible to reach these values at high IoU thresholds. In most common practical conditions where image resolution and object sizes are a lot smaller, an IoU of 0.5 like we used to compute the F1-score may even be too high to produce good results. In these cases, the smaller the bounding box, the more impact a few pixels off the target would have [24, 38]. This makes the standard mAP, as it is computed by default in MMDetection over high IoU thresholds, even worse suited for the task of counting animals in aerial images.

The mAP is a convenient metric for a general benchmark, as the quality of the localization of a prediction may be what differentiates a model from a slightly better one. However, this depends to what degree the correctness of the predicted bounding box is important compared to other aspects of the object detection task. In our case, the location of a deer is not as important as its detection. Therefore, mAP doesn’t seem well in line with the objectives we have when conducting aerial animal surveys. Considering the 10 different IoU thresholds over which are averaged the precision to produce the mAP, nine are higher than what was used during the inference. In this regard, the mAP is clearly focused on the quality of the localization of the prediction rather than purely on what we would consider good detections in an operational setup as is the F1-score.

The fact that the mAP is used as a training and validation metric in our setup may also have an impact on our objectives of producing accurate counts. By selecting the highest mAP during training, we may bias the selection towards better localization instead of fewer prediction errors. The exact same training may have resulted in selecting a model at another epoch if the validation metric had focused more on detection mistakes, like the F1-score would. Using the validation (or test) metric as a training metric has two benefits: it allows to tailor the model’s weight selection to the task and ensures that the performance is directly readable on the validation logs, instead of having to generate a different output once the training is complete.

In the context of animal surveys, the objective of trying different architectures is to select the one that is best at accurately counting animals. While the SCC between mAP and F1-score on our datasets shows a positive correlation between the two metrics, the discrepancies shown by GFL, VFNet and FRCNNX101 on both metrics is enough, in our eyes, to discourage the use of mAP to rank networks for such a specific purpose as animal counting. Without GFL, which outperformed every other model but FRCNNX101 on both metrics, we would have picked VFNet over FRCNNX101 based on its better mAP without realizing that we were selecting a worse performer on the counting task that we want to use it for. In our context it seems more relevant to select and evaluate models based on F1-score or another practical count-based metric and only use mAP as additional information to gauge the quality of the bounding box location. Moreover, we could also set a higher IoU threshold to compute the F1-score if we need a better location match between ground truth and predictions. In our case, FRCNNX101 and GFL performed similarly on F1-score but the location accuracy of the predictions for GFL, as seen through the mAP, was higher. It also has the possibility to deal with hard samples on the fly with its focal loss function. We would therefore select GFL over the rest of the models.

On our datasets, the newest model we tried (VFNet) performed worse on F1-score than its backbone alone (FRCNNX101). The specificity of VFNet’s architecture may have a role to play in this, as its loss has been specifically designed to improve the localization of the prediction’s bounding box. While we are aware that this could change with a thorough hyperparameter tuning, our experiments show that newer doesn’t always equate to better.

### 4.3 Imbalance

The impact of adding many negative images had the unexpected result of slightly improving the average performance. A possible explanation for this would be that the posOnly training images were lacking enough diversity of background to be able to correctly process some background in the unbalanced validation set. It would also mean that what we consider imbalance is not so in the eyes of the models. This would explain why models with no built-in focal loss, such as FRCNNR50 and FRCNNX101 still performed better on the wds datasets. Another surprise was that the most negatively affected network was GFL, which has the most advanced focal loss of the six. This may be corrected after a finetuning of the focal loss parameters.

In some cases, deer were missed at the confidence threshold of 0.5 but detected at a lower threshold. While the confidence threshold allowed to filter a large amount of false positives at the cost of some false negatives, leading to a higher F1-score, it would be beneficial for long term performance to increase the relative weight of these hard samples compared to the easier ones. This could be achieved by fine-tuning the focal losses of the models that have it or by a manual hard sample mining for models like FRCNN.

Focal loss requires to train on the whole dataset so that the hard samples can be processed on the fly. In a case of extreme imbalance such as in Peng et al. (2020), it would lead to very long training times where most samples would have a negligible impact. Hard Negative Mining (HNM) is a lot more efficient as it only selects the images with hard samples, leading to shorter training times [39]. Therefore, it seems like HNM and focal loss show an interesting synergy. While the first one selects hard samples to add to the training, the second one balances their importance during the training. False positives are objects that the network perceives as closer to the positive class than to the background at a given confidence threshold. The false positive class technique [4, 28] forces the network to learn the boundary between false positives and the objects of interest. In this regard, we believe it is a significant improvement to standard HNM methods. Because animal population management requires regular data acquisitions, the distinction between the positive class and the false positive class is naturally set to improve with time.

### 4.4 Background uniformity

In winter conditions, the improvement on mAP across all models was not transferred to the F1-score in the same magnitude, showing again that it is not a reliable predictor of counting performance. The mAP was considerably higher across all models in the winter dataset than on the summer acquisition. This difference appears to increase at higher IoU thresholds. A striking example of this is the best performer GFL, that had an AP of over 0.8 at an IoU of 0.9 on the winter datasets compared to close to 0.05 on both summer datasets. This shows that the localization of the predictions is a lot more accurate in the winter than on the summer acquisition. Our interpretation is that on the winter dataset, as the snow covered most of the areas around the animals, it improved the contrast between them and the background, reduced the difficulty of separating a deer from the rest of its environment and therefore led to better localization of the predictions. Having more similar conditions between training and validation sets also reduced the number of objects that could be misclassified as a deer, reducing the background intraclass diversity and with it the number of errors, leading to an increase of F1-score. However, the threshold of IoU of 0.5 seems to be forgiving enough to detect the animals in the summer acquisition and to obtain good counting performance as a result.

This gain in performance of both localization and counting accuracy makes acquisitions on homogenous terrain like snow very convenient as it reduces the overall difficulty of the detection task and leads to more consistent results. The winter dataset has more images of deer than its summer counterpart, which is likely to also contribute to the increased performance. However, we ran some training of up to 50 epochs on the summer dataset while evaluating the gains of longer training and never reached the levels of performance reached on the winter dataset. While more formal tests may be needed to confirm this, we believe the difference in background variability and increase in contrast between the deer and its surroundings contributed much more to the increase in performance of the models, especially along the mAP, than the number of samples in the dataset did.

Another important aspect to consider is the average impact of dataset quality (background uniformity and imbalance) compared to the average impact of network architecture. The dataset quality alone improved the performance from 0.94 on our most difficult dataset, S_posOnly, to 0.96 on our easier one, W_wds. Not only did dataset quality impact the average performance of our models, it seems it also amplified the effect of the different architectures: on S_posOnly, the standard deviation in F1-score is twice what it is on W_wds. This shows that considering dataset quality is paramount as it may have a bigger impact on the counting performance than the choice of architecture. This result seems to be in-line with a recent shift within the field of deep learning called ‘data-centric AI’. This approach moves the focus on improving data quality instead of trying to further improve the model’s architecture [40]. Therefore, we highly recommend practitioners to invest time thinking about the best ways to design the data acquisition campaigns and data processing steps to improve the overall quality of the datasets.

In a context of regular acquisitions of images over a designated area, we expect the majority of confusing objects to be learnt by the model over time in a way that could not be replicated in a one-time acquisition and test. The training and test set will likely become more similar and the number of false positives will naturally decrease leading to an improvement in the overall accuracy of the counts. This capacity of refining the detection capacities of the models overtime is in our eyes a major benefit of using deep learning to monitor animal populations.

### 4.5 Visualisation

**Fig 6:**
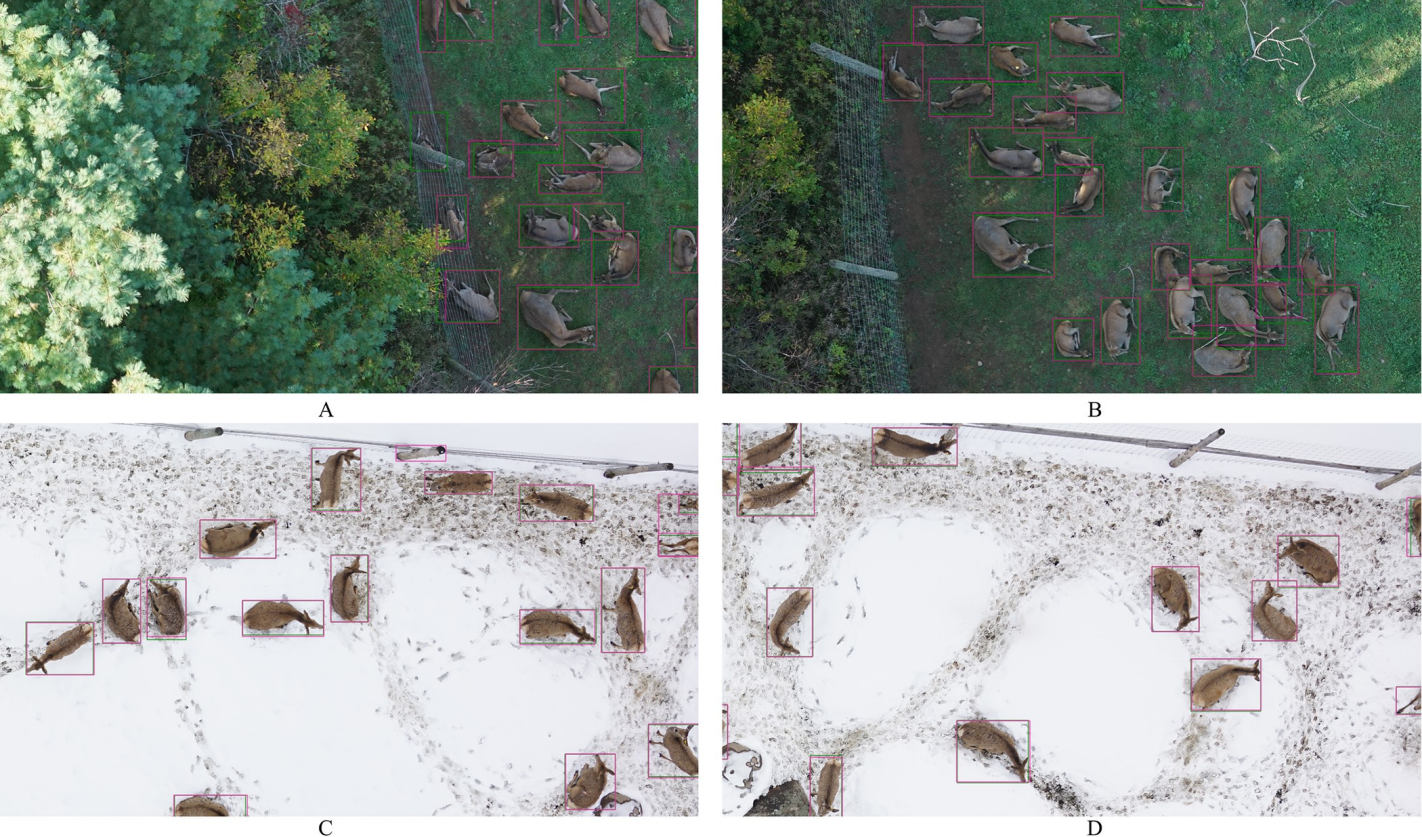
Example of prediction bounding boxes with mistakes (A and C) and without mistakes (B and D).

The overall quality of the predictions of our best performer was very good, with few mistakes (Fig 6).

**Fig 7:**
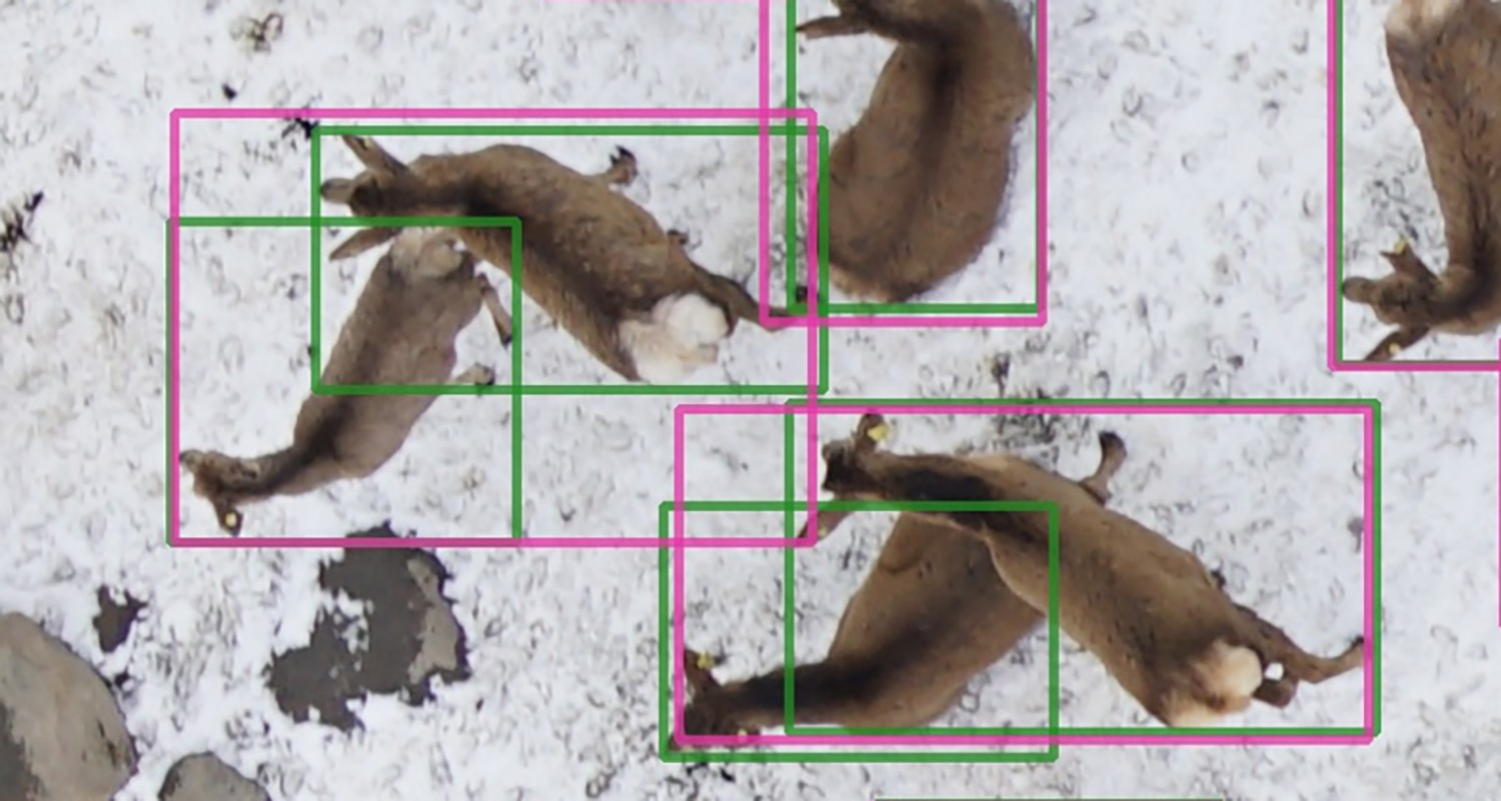
Example of prediction bounding boxes encompassing two ground truth bounding boxes, leading to up to one false positive and two false negatives.

However, through visual assessment of the predictions, it seemed it was having more difficulties drawing correct bounding boxes when two deer were very close or when parts of one deer would overlap with a second individual. It would then draw one large box encompassing the two deer, thereby counting as one or two misses and a false positive while it had considered both animals enough to include them into one large box (Figs 6C and 7). Peng et al. (2020) showed similar issues on their images. Adapting the size and aspect ratio of the bounding boxes based on the size of the objects in the training set has shown very good results. This may remove the cases where the prediction was much larger than a single deer but does not solve the issue entirely when two deer partially overlap. Another promising avenue would be through density maps, that seem to be particularly effective at dealing with partial overlaps between animals [41] and only require point annotations instead of the labor-intensive bounding box.

## 5. Conclusion

Our study showed that it is possible to obtain very good results in detecting animals in aerial images with little training time using pretrained models. With the number of different models available in MMDetection and its ease-of-use through prewritten training scripts and configuration files that still allow in-depth modifications, we believe it to be a valuable tool to quickly set up an automatic animal detection pipeline. However, the specificities of the evaluation metric need to match the goal of the project. In this regard, mAP isn’t as well suited as F1-score to select the most accurate models for counting animals in a wildlife survey. The particularities of the architectures also need to be taken into consideration, as some high performing models on one task may not be as good in our context. For future projects, we will opt for GFL as it performed the best on F1-score but had the possibility to finetune its sensitivity to hard samples through its focal loss, which our second-best performer FRCNNX101 doesn’t have. We see GFL’s higher localization accuracy as a welcomed bonus. The impact of imbalance needs to be carefully evaluated as our study shows that some imbalance can be beneficial. Lastly, data acquisition campaigns should be carried out over uniform background whenever possible, as it seems to increase performance.

### 5.1 Future work

Our future work will explore the impact of image resolution and slicing of images as a preprocessing step on counting performance.
